# Angioedema: A Life-threatening Complication of Tissue Plasminogen Activator

**DOI:** 10.7759/cureus.2392

**Published:** 2018-03-29

**Authors:** Muhammad Khalid, Majd Kanaa, Yazan Alkawaleet, Muhammad Talha Ayub

**Affiliations:** 1 Department of Internal Medicine, East Tennessee State University, Johnson City, USA; 2 Internal Medicine, John H Stroger J. Hospital of Cook County

**Keywords:** angioedema, angiotensin converting enzyme, t-pa (tissue plasminogen activator )

## Abstract

Angioedema is a localized, non-pitting, non-dependent, submucosal, and subcutaneous swelling resulting from the extravasation of fluid into the interstitium due to the increased production of plasma kinins and histamine. It can present with urticaria or anaphylaxis and is usually associated with angiotensin-converting enzyme inhibitors (ACEis), complement deficiencies, or the side effects of tissue plasminogen activator (tPA). Orolingual angioedema following tPA for acute ischemic stroke is a transient, self-resolving hemifacial swelling contralateral to neurological deficits that can rarely progress to the airway, compromising it and leading to a life-threatening situation if not managed promptly.

## Introduction

Tissue plasminogen activator (tPA)-induced angioedema is a self-limiting condition but can rarely be life-threatening, necessitating emergent intervention. We present a case of a 78-year-old female who developed orolingual angioedema, leading to severe airway compromise following tPA infusion for acute ischemic stroke.

## Case presentation

**Figure 1:**
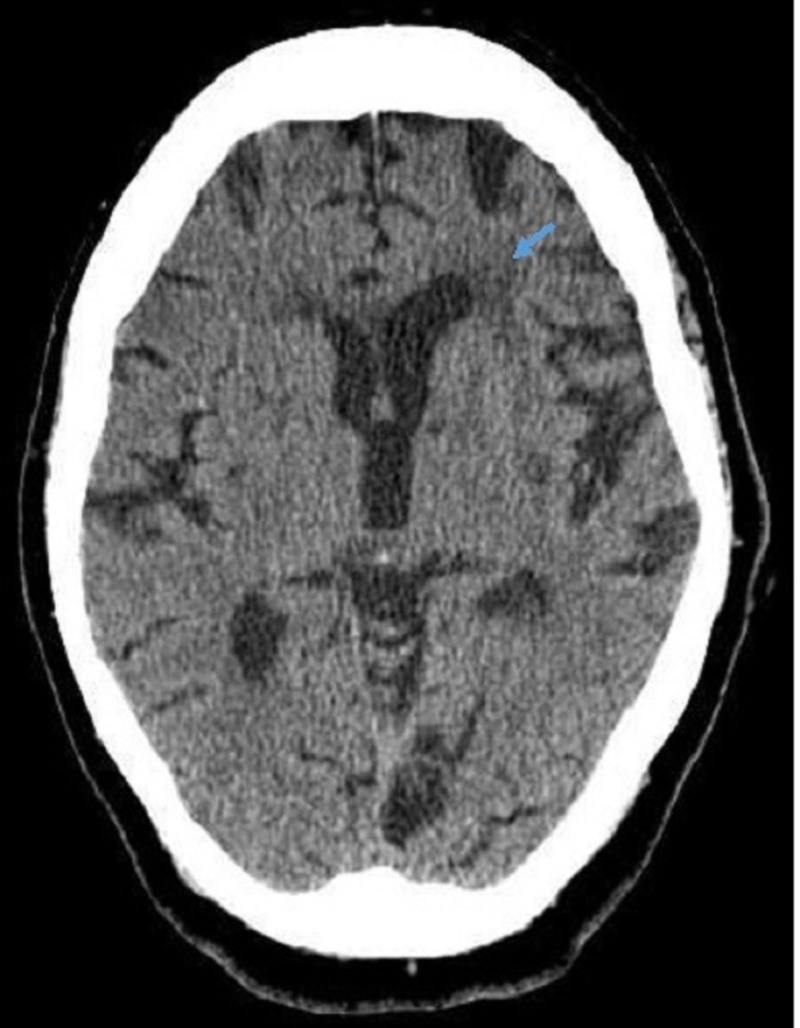
CT head showed an old infarct (blue arrow)

A 78-year-old female with a past medical history significant for hypertension, managed with angiotensin-converting enzyme inhibitor (ACEi), presented with complaints of aphasia, right hemiparesis, and facial droop from the last one hour. The patient noticed a sudden onset of right-sided weakness while getting ready for an appointment, and she could not put her clothing on and fell to the floor. Her daughter attempted to help her up, but she was unable to do so. The daughter noticed the patient had a right facial droop and aphasia. Her vitals on presentation were: blood pressure 202/68 mmHg, temperature 97.8 °F (36.6 °C), respiratory rate 14 per minute, and oxygen saturation 100% on room air. The physical examination was consistent with right-side hemiplegia, right facial droop, expressive aphasia, and dysarthria. Laboratory findings were unremarkable. Computed tomography (CT) scan of the head without contrast was not evident of any acute intracranial abnormality: hemorrhage, mass effect, midline shift, or hydrocephalus and showed an old infarct (Figure 1).

The patient was deemed a candidate for tissue plasminogen activator (tPA) for acute left-sided ischemic stroke based on the duration of symptoms and the absence of absolute contraindications. The patient was started on alteplase infusion (0.81 mg/kg) with close monitoring. The patient started complaining of dry mouth three hours later and was found to have progressively worsening tongue swelling, initially on the right side and then progressively on the left side. Alteplase infusion was stopped and she was managed conservatively with intravenous solu-medrol and diphenhydramine for a suspected allergic reaction. The patient had a progressive worsening of dyspnea and tongue swelling, requiring intubation and mechanical ventilation. The patient was extubated after a gradual improvement in tongue swelling following three days of intravenous steroids.

## Discussion

Orolingual angioedema is a life-threatening complication of the treatment of ischemic stroke with tissue plasminogen activator (tPA) with a reported incidence of 1-5% [1-2]. A multicenter study done by Hill et al. [1] reported angioedema in 15 out of 1135 patients (1.3%) treated with alteplase for ischemic stroke. Most of them were managed medically and overall results showed that stroke thrombolysis is an effective approach. A study done by Hill et al. [2] nine out of 176 (5.1%) patients treated with intravenous tPA for acute stroke were found to have orolingual angioedema and a relative risk of 13.6 with concomitant ACEi use and a relative risk of 9.1 with an ischemic infarct of the insula or anterior frontal cortex.

The most common dreadful complication of tPA is intracerebral hemorrhage reported in 2%-9% of the patients [3-4]. Angioedema is mostly seen in the setting of active angiotensin-converting enzyme inhibitor (ACEi) use but has been reported in patients without any exposure to ACEi [5-6]. A study done by Engelter et al. reported two out of 120 patients had angioedema after alteplase treatment in ischemic stroke. One out of 19 patients (5%) taking ACEi had orolingual angioedema compared to one out of 101 patients (1%) without taking ACEi [5]. Rudolf et al. reported two cases of angioedema in a cohort of 230 patients treated with alteplase for ischemic stroke [7]. A multicenter stroke registry by Lin et al. reported a lower incidence of alteplase-associated orolingual angioedema in the Asian population than in Caucasian and pre-stroke ACEi increased the risk of angioedema [8]. Another study by Hurford et al. reported that 42 out of 530 (7.9%) patients received alteplase treatment for ischemic stroke have orolingual angioedema and 172 (33%) were taking ACEi. Prior treatment with ACEi was an independent predictor of angioedema [9].

The pathophysiology of tPA-angioedema is related to bradykinin production by plasmin from the activation of plasminogen. Plasmin is a protease with fibrinolytic properties that resulted in the activation of the complement pathway by converting C1 to the active form, which, in turn, activates the rest of the complement cascade especially C3a and C5a, which release histamine and other inflammatory mediators, such as the kinin pathway. Normally, bradykinin is inactivated by kinase inhibitor but this pathway is inactivated in patients taking ACEi. It has a predilection for the orolingual area and has an asymmetric distribution contralateral to the side of the stroke. Increased vascular permeability as a result of bradykinin production from the breakdown of high molecular weight kininogen by plasmin, generated by tPA, is the mechanism of angioedema. The effect is more prominent in patients taking ACEI.

Angioedema is mostly self-limiting and can be managed conservatively by antihistamines and steroids. However, the rapid progression of edema leading to respiratory compromise needs emergent intubation to protect and maintain the airway. Our patient was on lisinopril for hypertension and developed rapidly progressive angioedema within three hours of the start of tPA infusion. Aggressive management with intubation to maintain the airway was life-saving after the patient failed to respond to standard medical therapy. The use of C1 esterase inhibitor and selective bradykinin inhibitor (icatibant) in tPA-induced angioedema refractory to conservative medical treatment has shown some promise [10].

A physician treating acute ischemic stroke with alteplase should be aware of this uncommon life-threatening complication and consider a routine inspection of the tongue and oropharynx for 30-45 minutes after the start of alteplase infusion and pay particular attention to patients taking ACEi. A history of ACEi use should always be obtained before starting treatment.

## Conclusions

Tissue plasminogen activator (tPA)-induced angioedema is a self-limiting condition but can rarely result in life-threatening airway compromise. There are no first-line treatment guidelines for angioedema caused by tPA in the treatment of ischemic stroke. Management includes antihistamines, epinephrine, and steroids. Emergent intubation is needed in cases of airway compromise due to the rapid progression and involvement of the larynx and hypopharynx despite medical treatment. The C1 esterase inhibitor should be considered an additional therapeutic option for patients with orolingual angioedema compromising the airway, which is refractory to the standard of care. Physicians should closely monitor for tPA-induced angioedema, especially in patients with ischemic stroke involving the frontal and insular cortex who are on angiotensin-converting-enzyme inhibitor (ACEi) therapy. The early recognition and cessation of thrombolytic therapy can prevent fatal consequences.
